# Effects of herbal medicine Sijunzi decoction on rabbits after relieving intestinal obstruction

**DOI:** 10.1590/1414-431X20176331

**Published:** 2017-09-21

**Authors:** L. Li, C. Zou, Z. Zhou, X. Yu

**Affiliations:** Department of Gastrointestinal Surgery, Tianjin Nankai Hospital, Tianjin, China

**Keywords:** Alternative medicine, Herbal medicine, Immune function, Intestinal obstruction, Nutritional status

## Abstract

Intestinal obstruction leads to blockage of the movement of intestinal contents. After relieving the obstruction, patients might still suffer with compromised immune function and nutritional deficiency. This study aimed to evaluate the effects of Sijunzi decoction on restoring the immune function and nutritional status after relieving the obstruction. Experimental rabbits (2.5±0.2 kg) were randomly divided into normal control group, 2-day intestinal obstruction group, 2-day natural recovery group, 4-day natural recovery group, 2-day treated group, and 4-day treated group. Sijunzi decoction was given twice a day to the treated groups. The concentration of markers was analyzed to evaluate the immune function and nutritional status. The concentration of interleukin-2, immunoglobulins and complement components of the treated groups were significantly higher than the natural recovery group (P<0.05). The levels of CD4^+^ and CD4^+^/CD8^+^ increased then decreased in the treated groups. The levels of tumor necrosis factor-α and CD8^+^ were significantly lower than the natural recovery group. The level of total protein in the treated groups also increased then decreased after relieving the obstruction. The levels of albumin, prealbumin and insulin-like growth factor-1 were significantly higher in the treated groups than in the natural recovery group (P<0.05). Transferrin level in the treated groups was significantly higher than the obstruction group (P<0.05). Sijunzi decoction can lessen the inflammatory response and improve the nutrition absorption after relieving the obstruction.

## Introduction

Intestinal obstruction is a challenging surgical emergency worldwide. Intestinal obstruction can be partial or complete and it normally occurs in the small or large intestine. Patients with intestinal obstruction suffer abdominal cramps, constipation, vomiting and bloating (1), and require decompression or surgical resection to relieve the intestinal obstruction. However, the abdominal surgery might cause recurrence of obstruction, which can be prevented by laparoscopic surgery (2). In addition, abdominal surgery leads to intestinal dysfunction with elevated morbidity and mortality.

The small intestine plays an important role in maintaining the normal physiological activities of vital organs. It is responsible for digestion, uptake and absorption of nutrients (3). Intestinal obstruction causes failure of the intestine functions, leading to an increase of intestinal toxins, high intestinal pressure, bacterial translocation and malnutrition (1). Consequently, intestinal obstruction will affect the immune system (4). After intestinal obstruction repair, patients might still have nutritional deficiency and compromised immune function.

Alvimopan is the common drug used for treatment of post-operative ileus but patients might experience side effects, such as nausea and vomiting (5,6). Sijunzi decoction is a traditional Chinese medicine that has been used for treatment of other diseases in previous studies as it has no side effects and it has a pharmacological effect in gastrointestinal function, immune system, ulcers and tissue repair (4,7,8). Sijunzi decoction, which is also known as the “Four Gentlemen” decoction, consists of four types of herbs: *Poria cocos*, *Radix ginseng*, *Glycyrrhiza uralensis* and *Atractylodes macrocephala* (9). The individual herbs in the decoction have been investigated and the active compounds were identified using LC/MS/MS as being ginsenoside, flavonoid and triterpenoid (10). The ratio of the different components in the decoction might affect the outcomes in different clinical indications. In this study, we aim to evaluate the effects of Sijunzi decoction on immune function and nutritional status after relieving the intestinal obstruction.

## Material and Methods

### Experimental animals

Healthy rabbits weighing 2.5±0.2 kg were provided by MingLe Experimental Animal Center, Tianjin. Animal studies were approved by the Animal Care and Ethical Committee of Tianjin Nankai Hospital. Thirty-six rabbits were randomly divided into normal control group, 2-day obstruction group (M), 2 days of natural recovery group (R1), 4 days of natural recovery group (R2), 2 days of treatment group (S1) and 4 days of treatment group (S2). Mechanical complete obstruction animal models were used. Briefly, scalpel, needle and thread were used to establish complete simple mechanical intestinal obstruction by the sigmoid ileal ligation method (11). After 48 h obstruction, small bowel obstruction was relieved by pumping 5 mL of normal saline.

After relief of obstruction, the treated groups were given 5 mL/kg of decoction twice a day. The Sijunzi decoction was prepared by mixing *P. cocos*, *A. macrocephala*, *R. ginseng* and *G. uralensis* in a ratio of 3:3:3:2. The mixture was boiled and concentrated to 1 g/mL. The decoction was sterilized and aliquoted, then stored at 4°C. Control and natural recovery groups were given normal saline twice a day. After 48 and 96 h of obstruction relief, rabbits were given 1 g/kg of urethane, 2 mL of blood samples were obtained and stored at −20°C.

### Immune function

Levels of interleukin-2 (IL-2) and tumor necrosis factor-α (TNF-α) were measured using enzyme-linked immunosorbent assay (ELISA; ADL Co., USA). Concentrations of IgG, IgM, IgA, and complement-C3 and C4 were measured using turbidimetric immunoassay (Beckman-Coulter Co., USA) (12). Levels of CD4^+^ and CD8^+^ T-lymphocytes were detected using flow cytometer (Antigenix America Co., USA). The Sijunzi treated group was compared with the M group and R2 group.

### Nutritional status

Blood samples were analyzed for total protein, albumin, prealbumin and transferrin at 48 and 96 h using MEGA bioanalyzer (Toshiba, Japan). Insulin-like growth factor-1 (IGF-1) assays were performed using radioimmunoassay after extracted by acid-ethanol method (Quest Diagnostics Nichols Institute, USA).

### Statistical analysis

Experimental data were analyzed using SPSS 20.0 (IBM, USA) and are reported as means±SD. Data between groups were compared using the *t*-test and one-way ANOVA. P<0.05 shows statistically significant differences.

## Results

### IL-2 and TNF-α

**Figure 1.**
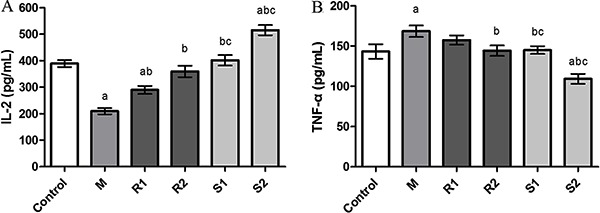
Levels of interleukin (IL)-2 (*A*) and tumor necrosis factor (TNF)-α (*B*) in rabbits with intestinal obstruction. M: intestinal obstruction group; R1: 2-day natural recovery group; R2: 4-day natural recovery group; S1: 2-day Sijunzi treated group; S2: 4-day Sijunzi treated group. Data are reported as means±SD. ^a^P<0.05 compared to normal control group; ^b^P<0.05 compared to M; ^c^P<0.05 compared to R1 and R2 (ANOVA).

Intestinal obstruction led to significant changes in IL-2 and TNF-α compared with the normal control group (Figure 1). After relieving the obstruction, IL-2 increased significantly and TNF-α of the R2 group decreased significantly (P<0.05).

### Immunoglobulins and complement components

**Figure 2.**
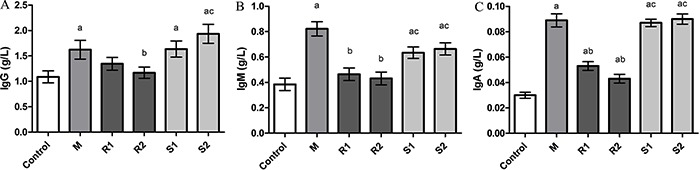
Concentrations of IgG (*A*), IgM (*B*) and IgA (*C*). M: intestinal obstruction group; R1: 2-day natural recovery group; R2: 4-day natural recovery group; S1: 2-day Sijunzi treated group; S2: 4-day Sijunzi treated group. Data are reported as means±SD. ^a^P<0.05 compared to normal control group; ^b^P<0.05 compared to M; ^c^P<0.05 compared to R1 and R2 (ANOVA).

**Figure 3.**
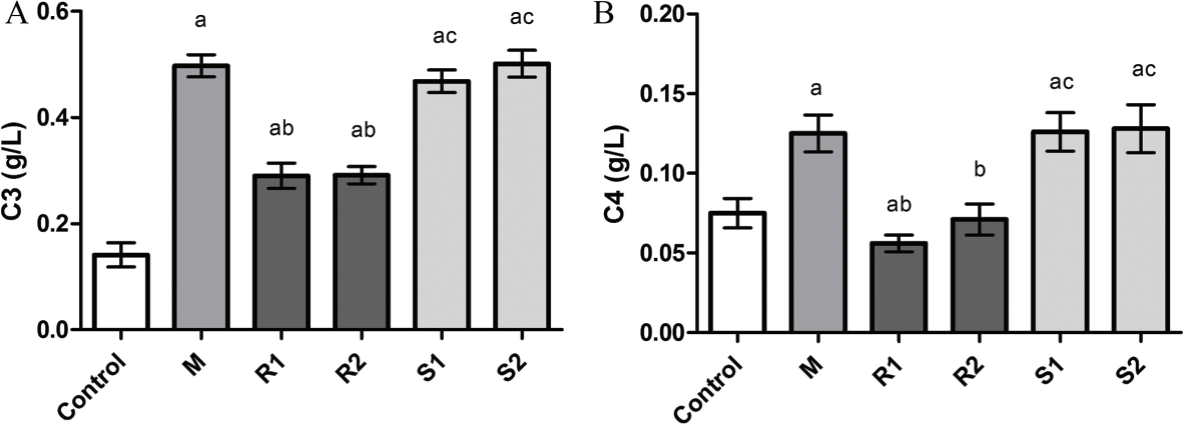
Levels of C3 (*A*) and C4 (*B*). M: intestinal obstruction group; R1: 2-day natural recovery group; R2: 4-day natural recovery group; S1: 2-day Sijunzi treated group; S2: 4-day Sijunzi treated group. Data are reported as means±SD. ^a^P<0.05 compared to normal control group; ^b^P<0.05 compared to M; ^c^P<0.05 compared to R1 and R2 (ANOVA).

Immunoglobulins and complement components were both significantly increased in the M group (P<0.05; Figures 2 and 3). After relieving the obstruction, immunoglobulins and complement components reduced significantly in the R2 group (P<0.05). Immunoglobulins and complement components in S2 were significantly higher than that of natural recovery group (P<0.05).

### CD4^+^ and CD8^+^ T-lymphocytes

Table 1 shows the flow cytometry results of CD4^+^, CD8^+^ and CD4^+^/CD8^+^ in each group. The CD4^+^ and the CD4^+^/CD8^+^ in the M group were significantly higher than the normal control group, while the CD8^+^ T-cell in the M group was significantly lower than the normal control group (P<0.05). After relieving the obstruction, CD4^+^ and CD4^+^/CD8^+^ in the natural recovery group were gradually reduced. After 4 days of natural recovery, CD4^+^ and CD4^+^/CD8^+^ were significantly lower than the normal control group (P<0.05), while CD8^+^ increased then decreased after relieving the obstruction. CD4^+^ and CD4^+^/CD8^+^ in the treated groups were significantly higher than the natural recovery group, while CD8^+^ was significantly lower than the natural recovery group (P<0.05).

**Table 1.** Flow cytometry results of CD4^+^, CD8^+^ and CD4^+^/CD8^+^.

Groups	CD4^+^ (%)	CD8^+^ (%)	CD4^+^/CD8^+^
Control	32.93±2.78	24.78±2.89	1.34±0.17
M	38.54±2.68^a^	21.49±0.73^a^	1.80±0.12^a^
R1	30.28±2.55^b^	26.48±1.86^b^	1.15±0.14^b^
S1	41.52±4.01^a^ ^c^	22.68±2.02^c^	1.89±0.29^a^ ^c^
R2	26.06±2.42^a^ ^b^	23.66±2.33	1.11±0.15^a^ ^b^
S2	36.07±3.23^c^	19.62±1.81^a^ ^c^	1.85±0.24^a^ ^c^

M: 2-day intestinal obstruction group; R1: 2-day natural recovery group; R2: 4-day natural recovery group; S1: 2-day treated group; S2: 4-day treated group. Data are reported as means±SD (n=6 per group).

a P<0.05 compared to control;

b P<0.05 compared to M group;

c P<0.05 compared to R1 and R2 (ANOVA).

### Nutritional status

**Figure 4.**
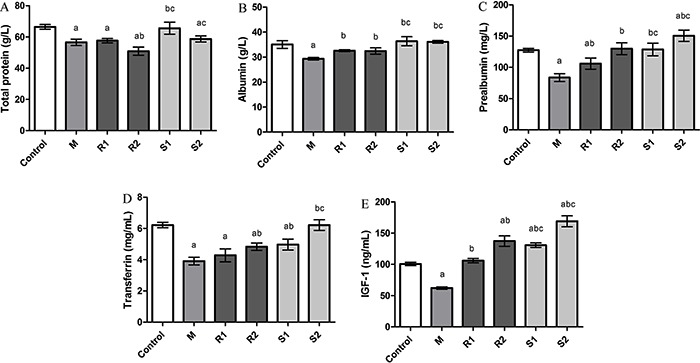
Levels of total protein (*A*), albumin (*B*), prealbumin (*C*), transferrin (*D*), and IGF-1 (*E*). M: intestinal obstruction group; R1: 2-day natural recovery group; R2: 4-day natural recovery group; S1: 2-day Sijunzi treated group; S2: 4-day Sijunzi treated group. Data are reported as means±SD. ^a^P<0.05 compared to normal control group; ^b^P<0.05 compared to M; ^c^P<0.05 compared to R1 and R2 (ANOVA).

All analyzed markers of nutritional status were reduced significantly in the M group (P<0.05; Figure 4). After relieving the obstruction, total protein in R2 reduced significantly (P<0.05; Figure 4A). However, the level of albumin, prealbumin, transferrin and IGF-1 increased gradually after relieving the obstruction. Application of Sijunzi decoction helped in increasing the levels of all markers. Total protein of the S1 group was significantly higher compared with the M group and R1 group (P<0.05). Total protein of S2 group decreased significantly compared to the normal control group (P<0.05). However, the total protein of the S2 group was higher than in the M and R2 groups. This might be due to the gastrointestinal function not being fully restored after relieving the obstruction.

## Discussion

According to traditional Chinese medicine theory, *Qi* is defined as a kind of psychophysical energy; disorder and stagnation of *Qi* and blood in organs will result in dysfunction of gastrointestinal motility (13). Therefore, Chinese medicine treatment focuses on promoting the flow of *Qi* and activates blood circulation in order to restore function of organs and clean them of toxins (14).

The four types of herbs in Sijunzi decoction have different functions: *R. ginseng* tonifies the *Qi* and strengthens the spleen and stomach; *A. macrocephala* strengthens the spleen, augments the *Qi* and drains excess fluids that affect the spleen and stomach function; *P. cocus* also strengthens the spleen and eliminates excess fluids; *G. uralensis* harmonizes the combination of herbs and moderates the draining properties of *P. cocus* (13). Sijunzi decoction can be used to treat many diseases, such as chronic gastritis, intestinal metaplasia, gastric and duodenal ulcer (15). It maintains normal bowel movement, digestion and absorption of nutrients in order to improve the nutritional status. Sijunzi decoction also regulates the gastrointestinal hormones and digestive enzymes to protect the gastrointestinal mucosa. A previous study showed that Sijunzi decoction helps gastrointestinal postoperative patients restore their immune function (16).

After relieving the intestinal obstruction, immune function was affected, however, the digestive system and nutrition absorption was not fully restored. Sijunzi decoction helps in regulating of a variety of immune factors and improves the nutritional status. This herbal medicine might be a more suitable alternative to drugs for restoring gastrointestinal function, as it does not cause adverse effects. Our experiments proved that Sijunzi decoction has the effect of replenishing *Qi* of the gastrointestinal organs, especially in the recovery of organ function after the removal of intestinal obstruction. Moreover, it has a good effect on removing the edema of the intestinal wall and tissue gap after intestinal obstruction.

### Effects of Sijunzi decoction on the immune function after relieving the intestinal obstruction

IL-2 was increased after relieving the intestinal obstruction, while TNF-α was decreased. IL-2 is a glycoprotein produced by T-lymphocytes and natural killer cells. IL-2 is important in the regulation of immune system that helps the T-cell proliferation and differentiation (17). IL-2 is also involved in the adjustment of immune response, enhancement of cytotoxic activity of CD8^+^ cells, and induces expression of the IL-2 receptor (18). IL-2 affects B-lymphocyte proliferation and antibody production, and induces proliferation and activity of natural killer cells (19,20). IL-2 promotes cytotoxicity of monocytes, and induces a variety of effector cells to secrete interferon-α, IL-4, TNF, colony stimulating factor and other cytokines (21). TNF-α is mainly secreted by monocytes and is an important cytokine in apoptosis pathogenesis, anti-tumor and anti-infection. An appropriate amount of TNF-α can stimulate T-cells and B-lymphocytes. However, an excessive amount of TNF-α will inhibit the immune system and promote activation of inflammatory mediators, which then results in tissue damage (22).

Significant reduction of immunoglobulins and complements after relieving the obstruction showed that the humoral immunity was compromised. CD4^+^ and CD4^+^/CD8^+^ were also reduced after relieving the intestinal obstruction. The CD8^+^ level increased and then decreased but was still significantly higher than after 4 days of intestinal obstruction relief. This shows that the cellular immunity was also compromised and that even after intestinal obstruction was relieved, the patient can still suffer from compromised immune function.

Previous research showed that Sijunzi decoction can enhance the T-lymphocyte activity and elevate the level of IgM in rats (23). After relief of the obstruction, immunoglobulins, complement components, CD4^+^ and CD4^+^/CD8^+^ of treated groups were significantly higher than the natural recovery groups. However, TNF-α and CD8^+^ of treated groups were significantly lower compared with the natural recovery groups. Application of Sijunzi decoction after relieving the intestinal obstruction can improve the immunity system and prevent infection and complications.

### Effects of Sijunzi decoction on the nutritional status after intestinal obstruction repair

In this study, all the markers of nutritional status except total protein showed significant improvement after relief of the obstruction. However, the levels of all markers except IGF-1 were lower than the normal control. This shows that intestinal obstruction affects the digestive system and the absorption of nutrients. After relieving the obstruction, the intestinal function slowly recovered and showed improvement, especially the level of IGF-1. Even though obstruction is relieved, the intestine might still have food intolerance and thus nutritional absorption will be affected. This may be related to the structure and function of the intestinal mucosa, as gastrointestinal motility is severely impaired (24). Therefore, treatment to restore the intestinal function is important for the patients after intestinal obstruction repair. IGF-1 is an important nutritional index.

In present study, Sijunzi decoction improved the nutritional status of rabbits after relieving the obstruction. Consumption of traditional Chinese medicine stimulates the secretion of gastric acid and proteases. It also stimulates hormones secreted by gastrointestinal mucosa cells, such as gastrin, cholecystokinin, and glucagon. These hormones promote gastrointestinal motility, increase blood flow and improve the physiological metabolic process. Sijunzi decoction increases the level of IGF-1, which stimulates growth of the intestinal mucosal barrier, upregulates the digestive enzymes, improves differentiation and maturation of immune cells, and improves the immune system (25).

Traditional Chinese medicine might be a more suitable alternative to Western drugs for restoring gastrointestinal function. Sijunzi decoction has a moderating effect on immune function. The specific regulatory mechanism is not entirely clear and requires further study. In future study, we might include low-dose, moderate-dose and high-dose groups.
